# Participation in a multicomponent lifestyle intervention for people with obesity improves glycated hemoglobin (HbA_1c_)

**DOI:** 10.3389/fcdhc.2023.1274388

**Published:** 2023-12-22

**Authors:** Mathias Høgsholt, Signe Kierkegaard-Brøchner, Ulla Milther Sørensen, Lene Bastrup Lange, Lene Sundahl Mortensen, Jens Meldgaard Bruun

**Affiliations:** ^1^ Department of Lifestyle Rehabilitation and Physio and Occupational Therapy, Horsens Regional Hospital, Horsens, Denmark; ^2^ Department of Medicine, Horsens Regional Hospital, Horsens, Denmark; ^3^ Department of Clinical Medicine, Aarhus University, Aarhus, Denmark; ^4^ Steno Diabetes Centre, Aarhus University Hospital, Aarhus, Denmark

**Keywords:** obesity, diabetes mellitus 2, glycated hemoglobin, weight loss, quality of life

## Abstract

**Introduction:**

Obesity is associated with compromised glucose metabolism. Hence, it is of interest to investigate if the lifestyle interventions used in the LIBRA-cohort, which aimed at not only weight loss, but also patient well-being, could also help obese patients improve glucose metabolism by evidence of reduced HbA_1c_. The aim of the study was to retrospectively investigate if patients who were referred to a lifestyle intervention for obesity, were able to alter HbA_1c_.

**Research design and methods:**

Patients with a BMI≥30 undergoing a 6-month lifestyle intervention, who also completed physical and mental health surveys and whose baseline and 6-month blood samples were available, were included in the analysis. For changes in HbA_1c_ and body weight a clinically relevant change of 5≥mmom/mol and 5%≥, respectively, was chosen. Participants were divided into groups according to their baseline HbA_1c_ level: “Diabetes”: HbA_1c_ of ≥6.5% (≥48 mmol/mol), “Prediabetes”: HbA_1c_ of 5.7% to 6.4% (39-47.99 mmol/mol) or “Normal” HbA_1c_ <5.7% (<39 mmol/mol).

**Results:**

180 patients met the stated inclusion criteria and these patients were divided into groups (median age (25^th^;75^th^ quartile): Diabetes: n=47, age 54 (43;60), 51% women, Prediabetes: n=68, age 60 (50;66), 71% women and Normal: n=65, median age 61 (50;66), 85% women. Significant reductions were found in all three groups and specifically in the diabetes group HbA_1c_ was reduced (mean [95%CI]) -5[-8;-2] mmol/mol from baseline to the end of the intervention. Furthermore, 35% of patients with prediabetes normalized their HbA_1c_ (<39) and 30% patients with diabetes reduced their HbA_1c_ <48. All groups had clinically relevant (≥5%) reductions in body weight (p<0.01). There was an association between body weight reduction and HbA_1c_ reduction in the diabetes group (p<0.01). All groups reported improvements in physical health (p<0.01).

**Conclusion:**

In this retrospective cohort study, all patients achieved clinically relevant weight loss after participation in the lifestyle intervention and obese patients with diabetes achieved clinically relevant reductions in HbA_1c_ after 6-months. More than 1/3 of patients with prediabetes normalized their HbA_1c_.

## Introduction

The International Diabetes Federation has estimated that more than 400 million people live with diabetes mellitus (DM) worldwide (1). In Denmark it is estimated, by using national registers, that approximately 280,000 people are living with DM, equivalent to 5% of the Danish population (2). The majority of these live with type 2 DM. A great number of people live with prediabetes, which is characterized by impaired fasting glucose levels typically without any symptoms. A study from 2018 estimated that 271,000 people in Denmark could be classified as having prediabetes, a condition associated with a higher risk of having a cardiovascular event and increased all-cause mortality (3). Furthermore, a study from 2017 indicated that people with a high glycated hemoglobin (HbA_1c_) level just below the threshold for diabetes had a higher risk of developing major adverse cardiovascular events than people in the diabetes range (4).

It has been established that the development of type 2 DM, is dependent not only on genetic factors but also on modifiable factors such as overweight and obesity, and a poorer lifestyle with physical inactivity and more unhealthy diet (5, 6). Hence, lifestyle changes may have a significant impact on the treatment of people with type 2 DM and prediabetes by lowering body weight, increasing levels of physical activity and improving diet, thereby attenuating metabolic risk factors such as HbA_1c_ levels (7, 8). Furthermore, in patients already using diabetes medication but with no further gain in HbA_1c_ reduction, a lifestyle intervention could be the key to reduce HbA_1c_ and hereby limit development of diabetes-related comorbidities.

Patient-reported outcomes serve as another important measure for evaluating treatment effects, especially health-related quality of life (HRQOL) (9, 10). Patients with DM have been reported to have lower self-reported HRQOL compared to healthy controls, and studies comparing changes in both HbA_1c_, body weight, and HRQOL in patients with obesity and DM or prediabetes are sparse (11, 12). Thus, the aim of this study was to investigate changes in, and associations between, HbA_1c_, body weight (kg), and HRQOL at baseline and to the end of the public lifestyle intervention (6 months). We hypothesized that patients with a high HbA_1c_ (≥48) would have a decrease in HbA_1c_ from baseline to 6 months.

## Methods

### Design

A prospective cohort study collected questionnaires from patients undergoing a lifestyle rehabilitation program at the Department of Lifestyle Rehabilitation, Braedstrup (13). We were permitted by the local hospital management to retrospectively access journals from the past 5 years to investigate blood samples and prescribed medication for the patients living in the Central Danish Region. The regional data protection agency regulated data handling (ID 1–16–02–704–20) and all data were stored in RedCap (14, 15) and MidtX (Alfresco CMS, version 1.30). Patients signed an informed consent form before inclusion.

### Patients

The study population consisted of patients with a BMI≥ 30, and who were admitted to the Department of Lifestyle Braedstrup with the goal of weight loss and improving well-being using a multifactorial lifestyle intervention (13). Patients were included in the cohort if they were able to read and understand the questionnaires. Patients having blood samples at baseline and at the end of the intervention (6 months) were included in the present study and grouped as “Diabetes”: HbA_1c_ of ≥6.5% (≥48 mmol/mol), “Prediabetes”: HbA_1c_ of 5.7% to 6.4% (39-47.99 mmol/mol) or “Normal”: HbA_1c_ <5.7% (<39 mmol/mol) according to the American Diabetes Association (ADA) (16). “Normal” refers to “normoglycemic”.

### Intervention

Independent of the HbA_1c_ level, the patients received the same intervention program. The intervention, as described in detail elsewhere (13), consisted of three modules of four days visits at the rehabilitation center during 6 months with phone calls in between. The lifestyle intervention had its focus on improving patients’ well-being using motivational interviews, situated learning and systemic coaching led by healthcare professionals. Individual counselling to recognize the patients’ individual focus areas, and in collaboration with a counsellor setting goals for the intervention period was executed. During their 4-day visits, patients were invited to take part in cooking meals with other patients and the kitchen staff. Furthermore, daily sessions of varying in- and outdoor physical activities were offered (hot pool, games, structured and purposeful exercises, and yoga). The activities were mainly group-based and led by healthcare professionals. Last, each patient had a healthcare professional as a contact person with whom they held individual conversations to facilitate the dialogue around personal and more sensitive issues during their 4-day visit. The contact person offered at least one telephone counselling between the 4-day visits.

### Data collection

#### Questionnaires

At baseline and the end of the intervention patients answered questionnaires regarding their self-reported body weight (kg). We considered a reduction in body weight of 5% to be clinically relevant (17). Furthermore, patients completed the Short-Form 12-Item Health Survey (SF-12) (18) measuring their physical and mental health.

The number of days the patients were moderately physically active (competitive or recreational sports, cycling with a moderate to fast pace, heavy gardening, power walking or physically demanding daytime work) for 30 minutes served as the patient’s physical activity level. Furthermore, patients completed questionnaires concerning their diet. The questionnaire consisted of 28 items in six categories (**Supplementary Table 1**, **Appendix**). On behalf of the patient’s intake of fruit, vegetables, fish and fat a composite score was determined and rated as either “healthy diet”, “medium diet” or “unhealthy diet”. The Centre for Research into Disease Prevention and Health in Copenhagen, Denmark developed the questionnaire (19) (Attached in Appendix).

#### Objective measures

Patients’ blood samples were retrospectively collected from baseline and at the end of the intervention. We considered a reduction in HbA_1c_ of 5 mmol/mol (0.5%) or more to be clinically relevant (20).

### Statistics

Data were presented as means and standard deviation (SD) given normal distribution of data, inspected by histograms and quantile-quantile-plots. If not normally distributed, continuous data were presented as median and 25^th^ and 75^th^ quartile. Changes in HbA_1c_, body mass and SF-12 all within the three groups were analyzed using t-tests reporting the mean change and 95% confidence interval when the changes were normally distributed. Associations between changes in HbA_1c_ and changes in body mass or SF-12 were investigated using regression statistics reporting the R^2^, coefficient with a 95% confidence interval (CI) and the p-level. The p-level was set at 0.05, and Stata 17 was used for the statistical tests (21).

## Results

**Figure 1:**
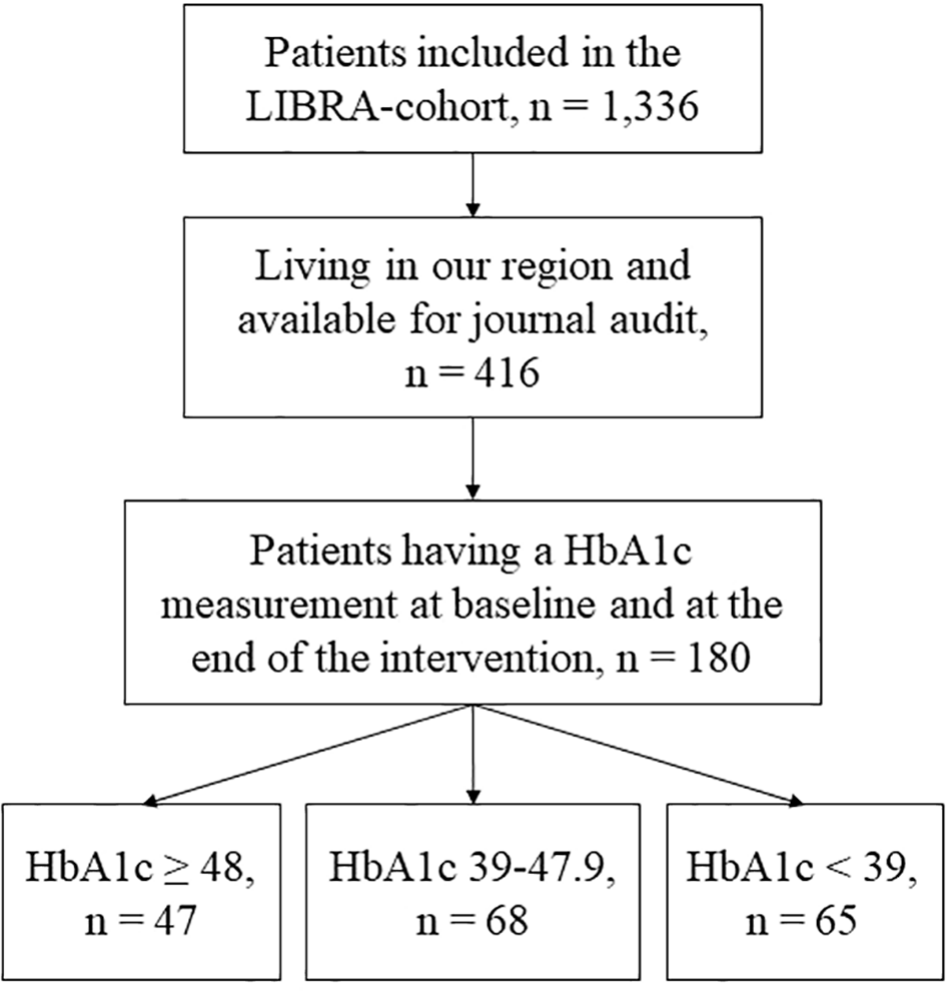
Flow chart.

Of 416 eligible patients a total of 180 patients were included in the study and their flow is seen in **Figure 1**. 97% of patients in the diabetes group, 42% of patients in the prediabetes group and 23% of patients in the normal group had prescribed diabetes medication.

Outcomes are presented in **Table 1**. All patient had significantly changes in HbA_1c_ and patients in the diabetes group had a clinically relevant change in their HbA_1c_. 35% (n=24) of patients with prediabetes normalized their HbA_1c_ (<39) and 30% patients (n=14) with diabetes reduced their HbA_1c_ <48. The mean percentage change in body weight as well as +/- standard deviation of each group was: “Diabetes”: -5 ± 6%, “Prediabetes”: -6 ± 5% and “Normal”: -5 ± 6%. SF-12 subscale scores can be found in **Supplementary Table 2**, **Appendix**.

**Table 1:** Outcomes.

	Baseline							End of intervention							Change baseline to end of intervention		
	Diabetes Median (25th;75th quartile) or % (n)		Prediabetes Median (25th;75th quartile) or % (n)		Normal Median (25th;75th quartile) or % (n)			Diabetes Median (25th;75th quartile) or %		Prediabetes Median (25th;75th quartile) or %		Normal Median (25th;75th quartile) or %			Diabetes Mean [95% CI] or p-value	Prediabetes Mean [95% CI] or p-value	Normal Mean [95% CI] or p-value
N	47		68		65												
Age	52	[43;60]	60	[50;66]	61		[50;66]										
% (n) women	51	(24)	71	(48)	85		(55)										
HbA_1c_ [mmol/mol]	58	[51;69]	42	[41;45]	36		[34;38]	52	[47;62]	41	[38;43]	35	[33;37]		-5[-8;-2]	-1[-2;-0.4]	-1[-1.5;-0.5]
% (n) patients with diabetes reduced to <48								30	(14)		–		–				
% (n) patients with prediabetes reduced to <39									–	35	(24)		–				
Body weight [kg]	115	[100;127]	112	[101;127]	108		[93;123]	107	[93;121]	104	[96;117]	102	[88;117]		-5[-7;-4]	-7[-8;-5]	-6[-8;-5]
SF-12 [0-100]																	
Physical health	33	[26;44]	37	[30;43]	41		[32;48]	40	[32;51]	42	[33;49]	44	[36;51]		4[2;7]	4[2;7]	4[1;6]
Mental health	47	[39;55]	48	[41;56]	46		[40;52]	45	[40;56]	52	[42;57]	52	[44;58]		1[-2;3]	2[-1;4]	4[1;7]
Unhealthy diet (%)		19	15		9				9		1		8		0.025	0.007	0.414
Days with PA (n)	3	[1;5]	3	[1;5]	3		[2;5]	3	[2;5]	3	[2;6]	4	[2;5]		0.123	0.028	0.121

PA, Physical activity.

While all groups self-reported experiencing an increase in physical health only the “normal” group, self-reported experiencing an increase in their mental health.

**Figure 2:**
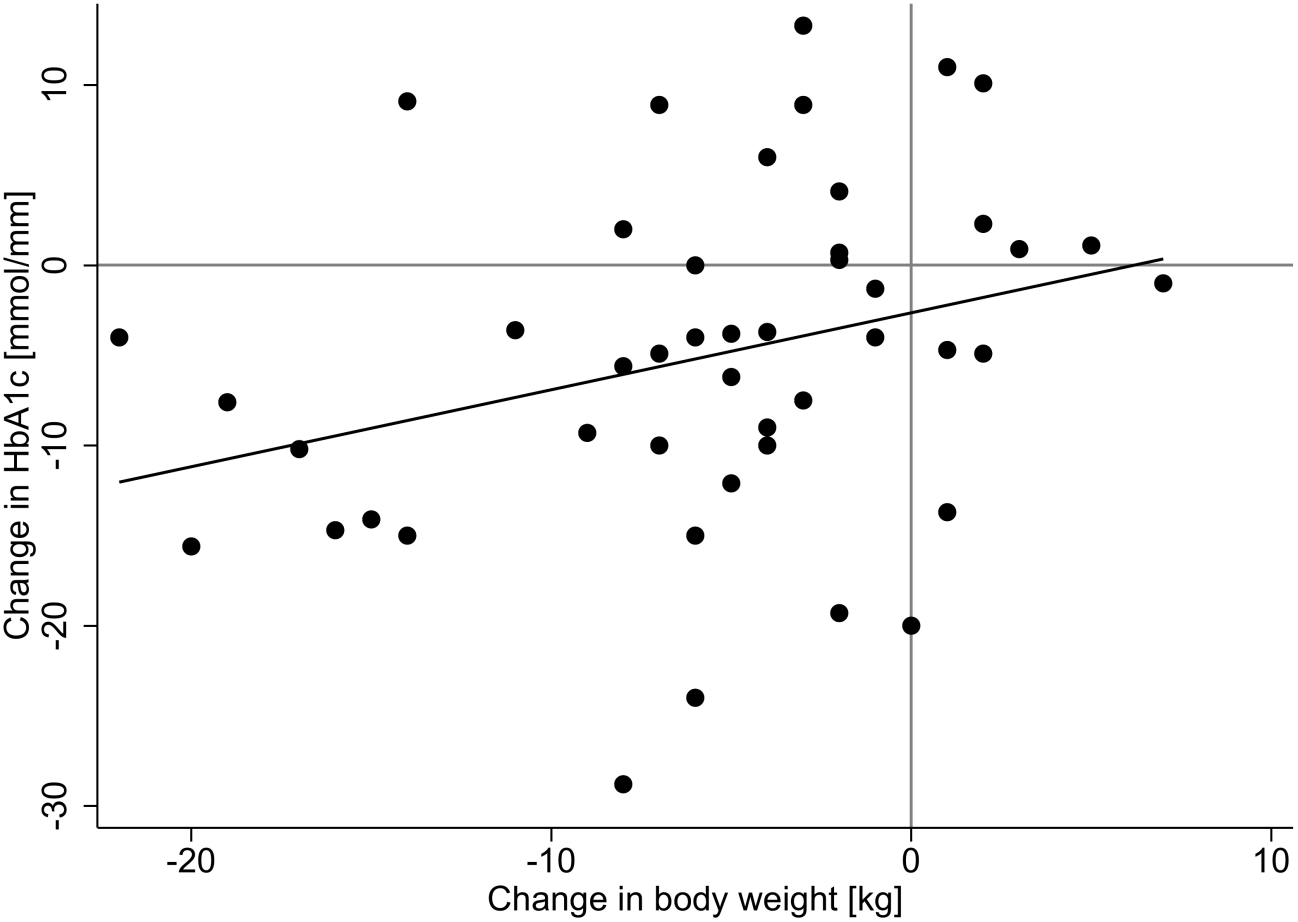
Scatter plot.

We found a weak (R^2^ = 0.07-0.09), but significant association (**Table 2**) between weight reduction and HbA1c reduction in the diabetes and prediabetes group (**Figure 2**).

**Table 2:** Associations of body weight changes to HbA_1c_, and Physical/Mental health.

		Change in body weight			
		R^2^	Coefficient (mean [95%CI])		p-value
**Change in HbA_1c_**	Diabetes	0.09	0.43	[0.02;0.84]	0.042
	Prediabetes	0.07	0.15	[0.02;0.28]	0.029
	Normal	0.04	0.06	[-0.01;0.13]	0.108
**Change in physical quality of life**	Diabetes	0.10	-0.36	[-0.71;-0.004]	0.048
	Prediabetes	0.23	-0.66	[-0.98;-0.34]	<0.001
	Normal	0.01	-0.12	[-0.52;0.28]	0.547
**Change in mental quality of life**	Diabetes	0.01	0.13	[-0.27;0.52]	0.520
	Prediabetes	0.003	-0.08	[-0.49;0.33]	0.706
	Normal	0.06	-0.41	[-0.87;0.05]	0.081

R, regression coefficient; CI, confidence interval.

## Discussion

In this retrospective study, we found that patients with obesity participating in a 6-month lifestyle intervention experienced clinically relevant improvements in HbA_1c_ alongside a clinically relevant decrease in body weight. Furthermore, positive outcomes where seen for patients with prediabetes where 35% normalized their HbA_1c_ after the intervention.

Glycemic control is of great interest for both type 1 and 2 diabetes to reduce the risk of both micro- and cardiovascular complications (22). Reduction of HbA_1c_ is essential as the risk of diabetes-related complications is reduced when reducing HbA_1c_ (23). One of the primary aims of the clinical practice is to reduce HbA_1c_ and hereby reduce the amount of diabetes-related complications. Hence, it is of great clinical relevance, that HbA_1c_ was reduced with the current lifestyle rehabilitation program. Other studies have investigated patients with altered glucose metabolism. In a study from the United States, Knowler et al. (24) investigated patients with prediabetes having either metformin or a lifestyle intervention. Both groups reduced their incidence of diabetes at mean 2.8 years follow up, but with the largest effect in the group receiving a lifestyle intervention. Our patients with prediabetes had a stable, slightly decrease in HbA_1c_ and 35% of the patients with prediabetes had normalized their HbA_1c_ at the end of the intervention. Hence, participation in a lifestyle intervention in both studies seems of value for patients with prediabetes. In another study from United States, Delahanty et al. (25) investigated two types of lifestyle interventions in patients with diabetes, a BMI≥25 and 83% using diabetes medication. The authors found similar reductions in HbA_1c_ as in the current study together with a similar weight loss. Hence, across different populations and geographical locations lifestyle interventions had favorable outcomes for patients with prediabetes and diabetes and should be in the pool of treatment choices for patients when diagnosed.

While the post physical health scores were lower in the diabetes group, 40, when compared to the normal group, 44, this was not seen as significant as the change from baseline was the same for both groups (+4). It would have been in good correspondence with patients with diabetes being more physically impaired than patients without diabetes. On the other hand, all patients had a BMI ≥30, hence, some of them could be limited in physical function by their body weight, especially those being very obese who struggle being physically active due to their larger body (26). All patients improved in their physical health score which could be partly related to their weight loss. We saw that there was a weak but significant association between weight loss and improvement in physical health score. Hence, improvement in weight explained some of the variation but multiple other unknown factors affected the variation.

Only the normal group experienced a change in mental health score. However, all groups had initially high mental health scores (approaching 50 points) with corresponding lower degrees of possible improvement. The SF-12 scores are adjusted to a population mean of 50. Hence, when the patients have a mean of 50, it corresponds to patients having a similar mental health as the general population and further improvements are, hence, less likely. We are therefore not unsatisfied with these results.

The diabetes and prediabetes groups had a reduction in the percentage of patients eating an unhealthy diet. In this study “unhealthy” diet was defined according to a questionnaire used in the Danish population-based study “*How are you feeling?*” (19). We were not involved in selecting what comprises an unhealthy diet. In the earlier published cohort study (13), we problematize that it seems that a smaller amount of the patients reported an unhealthy diet than expected – e.g. we saw an underreporting of intake of cake, sugar and likewise (social desirability bias). Hence, in future studies, the validity of the diet questionnaire in this patient group should be investigated.

The present study is limited by its retrospective design. Only patients who had HbA_1c_ measured both pre and post intervention were included in this analysis. This reduced our cohort from a possible 416 to 180 (please, see **Figure 1** for details). There could be many reasons for missing the blood samples and some of those severely bias the estimates, especially concerning lack of mental or physical resources to attend an outpatient clinic or general practitioner having blood samples taken. The mental health in the three groups were approaching the mental health of the general population, hence, the included patients in this study could have higher mental resources than other parts of the population. However, if we compare mental health scores in the study group of patients who attended blood samples with the total cohort of 1,336 patients, baseline mental health scores were close (1-2 points apart) (13). Hence, we are not worried that only the subset of patients with high mental capacity attended the blood samples. However, other factors could have affected the sample as it was retrospectively collected. Hence, the findings of the present study should be replicated in a prospective study before certainty of the results can be evaluated. Another limitation is that we could only collect data regarding prescribed medication and not the development of the use of medication. This should be further investigated in a future study.

## Conclusion

In this retrospective study, patients with obesity and type 2 diabetes experienced a clinically relevant reduction in HbA_1c_ after participation in a 6-month lifestyle intervention focusing at multiple components. Additionally, 35% of patients with prediabetes normalized their HbA_1c_ after participation in the intervention. Last, the mean patient across all groups obtained a clinically relevant body weight loss ≥5% alongside improvements in physical health. All these positive effects were only partly associated with weight loss indicating that focus for a lifestyle intervention should comprise more than only weight loss.

## Data availability statement

The original contributions presented in the study are included in the article/**Supplementary Materials**. Further inquiries can be directed to the corresponding author.

## Ethics statement

The study involving human participants was reviewed and approved by the Committee on Biomedical Research for the Middle Region of Denmark. Handling of data was approved by the Data Protection Agency of the Middle Region of Denmark (ID: ID 1–16–02–704–20). Data for blood samples were collected retrospectively. Hence, in order to obtain these data, we had approval from the Hospital Management, Horsens Hospital, Denmark. The study was conducted in accordance with the local legislation and institutional requirements in Denmark. The patients provided written informed consent to participate in this study.

## Author contributions

MH: Formal analysis, Investigation, Methodology, Project administration, Validation, Writing – original draft. SK-B: Conceptualization, Formal analysis, Investigation, Methodology, Project administration, Software, Validation, Visualization, Writing – original draft. US: Conceptualization, Methodology, Validation, Writing – review & editing. LL: Conceptualization, Resources, Supervision, Writing – review & editing. LM: Conceptualization, Funding acquisition, Methodology, Resources, Supervision, Validation, Writing – review & editing. JB: Conceptualization, Investigation, Methodology, Supervision, Validation, Writing – review & editing.
