# Supplementary material for: Participation in a multicomponent lifestyle intervention for people with obesity improves glycated hemoglobin (HbA1c)
Source: Front Clin Diabetes Healthc. 2023 Dec 22;4:1274388. doi: 10.3389/fcdhc.2023.1274388 (PMC10766760; doi:10.3389/fcdhc.2023.1274388)
Supplement: Supplementary file 1 [file Table_1.docx]

**Appendix**

**Supplementary Table 1: Diet questionnaire**

Categories patients are ask about their intake of in the diet questionnaire:

| **On bread** | Butter |
| --- | --- |
|  | Vegetable margarine |
|  | Fat |
|  | Bread without fat |
|  |  |
| **For cooking** | Margarine for frying |
|  | Vegetable margarine |
|  | Butter |
|  | Fat/vegetable fat |
|  | Olive oil |
|  | Corn, sunflower oil |
|  | Rapeseed oil |
|  | Food without added fat |
|  |  |
| **Cold cuts** | Cold meats |
|  | Fish |
|  | Eggs |
|  | Mayonnaise-based salads e.g. chicken salad, tuna salad etc. |
|  |  |
| **Hot meals** | Meat |
|  | Poultry |
|  | Fish |
|  | Vegetarian |
|  |  |
| **Vegetables/greens** | Mixed green salad |
|  | Other raw vegetables |
|  | Cooked vegetables |
|  |  |
| **Sugary/fatty snacks** | Cake, sweets, ice cream |
|  | Crisps, popcorn |
|  | Fast food (pizza, burgers…) |
|  | Soft drinks/soda, squash/cordial etc. |
|  | Fruit |

**Supplementary Table 2**: SF-12 subscale scores

|  | **Baseline** | | | | | | **End of intervention** | | | | | | **Change baseline to end of intervention** | | |
| --- | --- | --- | --- | --- | --- | --- | --- | --- | --- | --- | --- | --- | --- | --- | --- |
|  | Diabetes | | Prediabetes | | Normal | | Diabetes | | Prediabetes | | Normal | | Diabetes | Prediabetes | Normal |
|  | Median (25th;75th quartile) | | Median (25th;75th quartile) | | Median (25th;75th quartile) | | Median (25th;75th quartile) | | Median (25th;75th quartile) | | Median (25th;75th quartile) | | Mean  [95% CI] | Mean  [95% CI] | Mean  [95% CI] |
| **Short Form-12 Subscale**  **Scores [0-100]** | | | |  |  |  |  |  |  |  |  |  |  |  |  |
| Physical Functioning | 28 | [28;47] | 37 | [28;47] | 37 | [28;47] | 37 | [28;47] | 42 | [28;47] | 47 | [37;47] | 3.2 [0.6;5.8] | 2.8 [0.4;5.1] | 2.6 [-0.3;5.5] |
| Physical Role | 39 | [29;48] | 39 | [29;48] | 39 | [32;48] | 39 | [34;48] | 43 | [39;53] | 48 | [39;58] | 2.1 [-0.5;4.7] | 4.4 [1.8;6.9] | 5.2 [2.6;7.9] |
| Bodily Pain | 30 | [30;48] | 39 | [30;48] | 39 | [30;48] | 39 | [30;58] | 39 | [30;48] | 48 | [30;48] | 2.4 [-1.0;5.8] | 3.1 [0.7;5.5] | 3.5 [0.9;6.1] |
| General Health | 32 | [32;46] | 32 | [32;46] | 32 | [32;46] | 46 | [32;46] | 46 | [39;46] | 46 | [32;57] | 5.0 [1.8;8.3] | 6.3 [4.0;8.6] | 6.0 [3.5;8.5] |
| Vitality | 39 | [29;48] | 39 | [39;48] | 39 | [39;48] | 48 | [39;48] | 48 | [39;58] | 48 | [39;58] | 2.8 [-0.1;5.8] | 3.8 [1.4;6.2] | 4.8 [2.0;7.5] |
| Social Functioning | 46 | [35;56] | 46 | [35;56] | 46 | [35;56] | 46 | [35;56] | 56 | [46;56] | 46 | [46;56] | 4.3 [1.4;7.3] | 2.1 [-0.2;4.4] | 4.2 [1.4;7.0] |
| Emotional Role | 42 | [32;52] | 47 | [37;58] | 47 | [37;58) | 42 | [32;52] | 47 | [37;58] | 47 | [37;58] | -1.0 [-4.2;2.2] | 1.3 [-1.5;4.1] | 2.8 [-0.3;6.0] |
| Mental Health | 46 | [40;52] | 46 | [40;52] | 40 | [40;46] | 46 | [40;52] | 46 | [40;52] | 46 | [40;58] | 1.0 [-1.5;3.6] | 2.3 [-0.3;5.0] | 4.2 [1.3;7.0] |

CI: confidence interval
